# Design principles for single standing nanowire solar cells: going beyond the planar efficiency limits

**DOI:** 10.1038/srep04915

**Published:** 2014-05-09

**Authors:** Yang Zeng, Qinghao Ye, Wenzhong Shen

**Affiliations:** 1 Institute of Solar Energy, and Key Laboratory of Artificial Structures and Quantum Control (Ministry of Education), Department of Physics, Shanghai Jiao Tong University, Shanghai 200240, People's Republic of China

## Abstract

Semiconductor nanowires (NWs) have long been used in photovoltaic applications but restricted to approaching the fundamental efficiency limits of the planar devices with less material. However, recent researches on standing NWs have started to reveal their potential of surpassing these limits when their unique optical property is utilized in novel manners. Here, we present a theoretical guideline for maximizing the conversion efficiency of a single standing NW cell based on a detailed study of its optical absorption mechanism. Under normal incidence, a standing NW behaves as a dielectric resonator antenna, and its optical cross-section shows its maximum when the lowest hybrid mode (HE_11δ_) is excited along with the presence of a back-reflector. The promotion of the cell efficiency beyond the planar limits is attributed to two effects: the built-in concentration caused by the enlarged optical cross-section, and the shifting of the absorption front resulted from the excited mode profile. By choosing an optimal NW radius to support the HE_11δ_ mode within the main absorption spectrum, we demonstrate a relative conversion-efficiency enhancement of 33% above the planar cell limit on the exemplary a-Si solar cells. This work has provided a new basis for designing and analyzing standing NW based solar cells.

Over the past decade, semiconductor nanowires (NWs) have been actively investigated as building blocks for third-generation photovoltaics^1^,^2^. Their remarkable advantages of reflection-reduction, light-trapping, and material-saving have proved critical for achieving high-efficiency solar cells at low cost. Despite all the merits, the existing NW-based solar cells are still restricted by the same fundamental efficiency limits as are the planar devices. These limits originate from the basic processes of solar energy conversion and have been concluded to be beyond the scope of the NW applications^1^. The conclusion, however, has been challenged by a few latest researches based on standing NWs. Krogstrup *et al.*^3^ have reported an absorption cross-section ~8 times larger than the geometrical cross-section on a single standing GaAs NW, which may function as a built-in light concentration and increase the open-circuit voltage beyond the planar limit. Another work by Wallentin *et al.*^4^ has indeed demonstrated a higher-than-planar *V*_oc_ on a sparsely distributed InP NW array, although its origin remains to be determined amongst several different possibilities.

Clearly, certain properties of the standing NW structure may hold the key to breaking the planar efficiency limits when utilized in novel manners. In the above cases, the Shockley-Queisser limit^5^ of cell efficiency—a fundamental limit imposed by the detailed balance condition of the cell-sun system—is notably breached by the much enlarged optical cross-section of the standing NWs. This effect has not been proposed on the previously well studied lying NWs because their optical cross-section is only about twice their geometrical ones^6^,^7^. Another potential of the standing NWs is to resolve the intrinsic current-losses in the defect-rich regions of the cell by utilizing its unique resonant features to shift the absorption front to a more ideal absorber within the cell. If realized, such effect would not only promote the cell efficiency beyond the planar structure limit, but also loosen the constraints on the critical parameters such as the doping concentration and the emitter thickness in cell designs. At present, the biggest obstacle to the quantitative analysis of all these possibilities is the lack of a theoretical description for the light-absorption mechanism within a single standing NW. Compared to the lying NWs, a standing NW is essentially finite in length, and its resonant modes cannot be accurately predicted by the previous methods based on infinite-waveguide analogies^6^,^7^.

In this work, we present a detailed investigation on the potential of the single standing NW structure to go beyond the fundamental efficiency limits that confine a planar solar cell. As the core concept, the optical absorption mechanism has been determined by employing the theories of finite-sized dielectric resonator antennas (DRAs)^8^,^9^, where we find that a single standing NW behaves exactly as a DRA under normal incidence and the enlargement of its optical cross-section strongly depends on the excitation of the lowest hybrid mode (HE_11δ_) due to the optical antenna effect. Based on this knowledge, we quantitatively examined the aforementioned effects for the exemplary a-Si solar cells. First, a *V*_oc_-increment of 124 mV is achieved by choosing an optimal NW diameter that supports the strongest HE_11δ_ resonant mode within the main absorption spectrum and leads to a maximal built-in concentration of 21-fold. Second, a notable rise in the internal quantum efficiency (IQE)—from 86% of the planar cell to 96% of the standing NW one—is observed when the primary absorption region is shifted to the intrinsic middle layer of the cell. This corresponds to a 3.5-fold reduction in the recombination-induced current-loss and exhibits an inherent tolerance to defects. Finally, a remarkably high conversion efficiency of 17.67% is resulted for the a-Si standing NW cell, showing a 33% relative increase over the planar cell limit. These observations have provided guidelines for designing and analyzing standing NW based solar cells and inspire novel methods in improving the cell efficiency beyond the planar efficiency limits.

## Results

### Open-circuit voltage beyond the planar limit

The most significant difference between the optical property of a sub-wavelength dielectric particle and that of a macro-scale one is that the former possesses a scattering/absorption cross-section larger than its geometrical one^10^. In the field of photovoltaics, such phenomenon was first observed and analyzed on lying NWs^6^,^7^,^11^, but has recently been demonstrated as even more pronounced on standing NWs^3^. In the latter case, a standing NW has two sub-wavelength dimensions parallel to the wavefront (whereas a lying NW has only one) and thus behaves as a more effective scatterer to light. Its optical cross-sections may extend to several times as large as its geometrical area^3^, which leads to an effective built-in light concentration.

**Figure 1:**
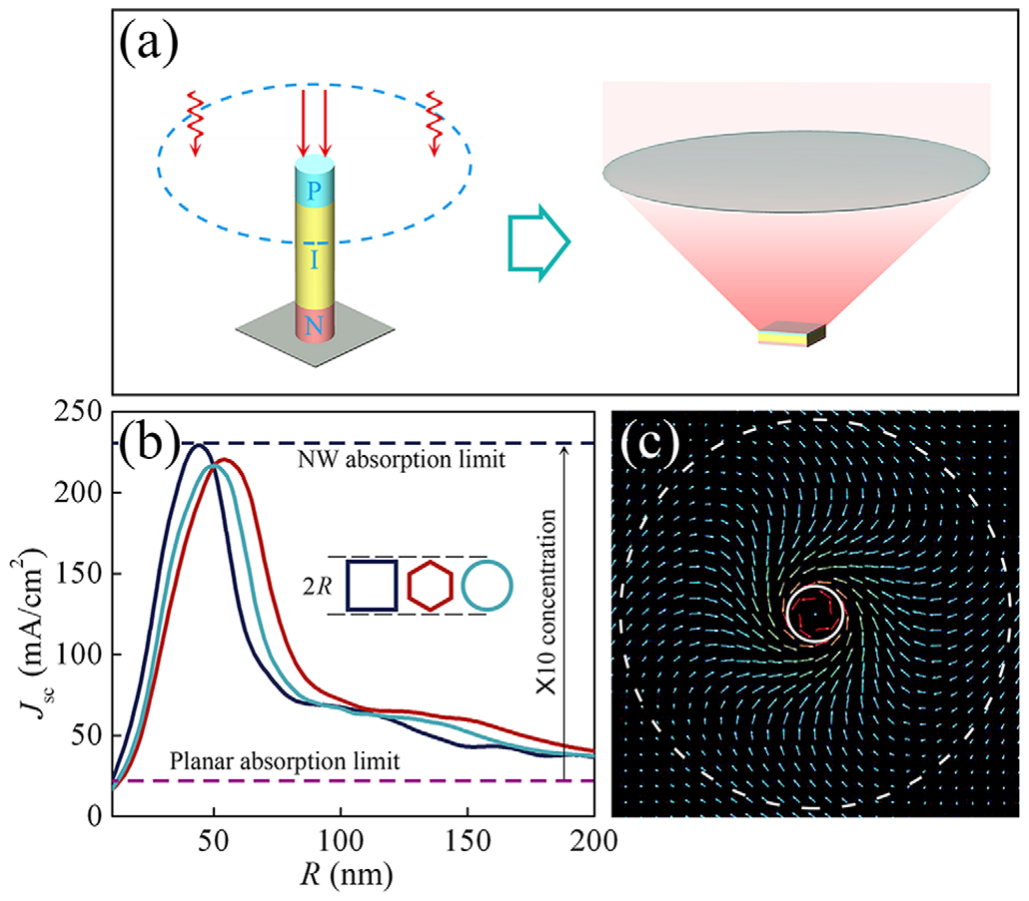
(a) Schematic 3D drawings of the single standing NW cell (the front and rear contacts are omitted) and the equivalent built-in concentration effect. (b) *J*_sc_ versus NW radius *R* for different NW morphologies under AM1.5 solar spectrum. The NWs are 500 nm in length on a transparent substrate. (c) Poynting vector in the middle plane normal to the NW axis. The solid and dashed white circles indicate the NW geometry and the approximate location where the Poynting vector directs away from the NW, respectively.

In Fig. 1(a), a schematic drawing illustrates the built-in concentration effect. A standing NW can not only absorb incident light within its geometrical boundary (indicated by straight arrows), but also absorb light further away from its axis (indicated by curved arrows). Thus it equals to a lens with a concentration factor *C* applied to a planar cell. Since the photocurrent density *J*_sc_ is proportional to the absorption cross-section while the saturation current density *J*_0_ remains constant, the *V*_oc_ could be improved by a factor of *nkT*ln*C* according to the formula *V*_oc_ = (*nkT*/*q*)ln(*J*_sc_/*J*_0_) (where *n* is the diode quality factor, *k* is the Boltzmann constant, *T* is the absolute temperature, and *q* is the elemental charge). In Fig. 1(b), we plot the *J*_sc_ of a standing NW on a transparent substrate (without a back-reflector) as a function of NW size for different NW morphologies (assuming an IQE of unit, under AM1.5 solar spectrum). The *J*_sc_ exhibits a significant enhancement over a wide range of NW size, especially for radius *R* ~ 50 nm where a 10 fold increase over the planar absorption limit is observed. The enlarged absorption cross-section comes from the well-known optical antenna effect, where the resonant modes supported by a sub-wavelength structure extend noticeably outside of its geometrical boundary and thus become easily exited by outer light-field^6^,^7^,^12^. A more intuitive image is shown in Fig. 1(c). It shows the Poynting vector in the middle plane normal to the NW axis, with the solid circle indicating the NW geometry and the dashed circle indicating the approximate location where the Poynting vector directs away from the NW. Apparently, external light energy can couple into the leaky modes of the NW even at places several radius away from the NW center, thus leading to an effective built-in concentration.

**Figure 2:**
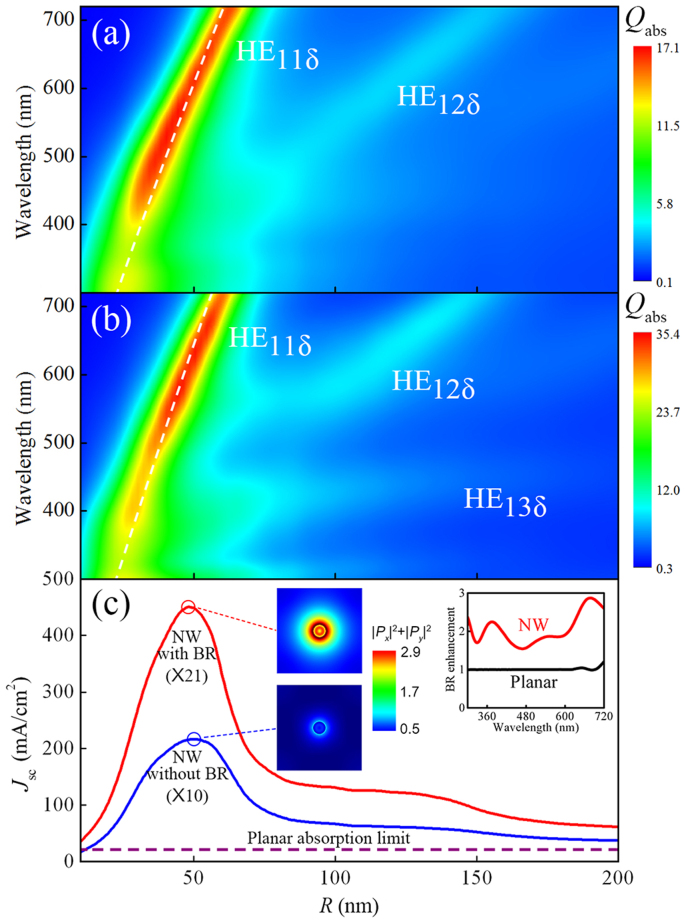
(a) and (b) Absorption efficiency *Q*_abs_ as a function of wavelength *λ* and radius *R* for NWs on a transparent substrate and on a perfect back-reflector, respectively. The dashed white lines indicate the calculated HE_11δ_ modes for isolated DRAs of length 500 nm and 1000 nm, respectively. (c) *J*_sc_ versus *R* for NWs with and without a back-reflector under AM1.5 solar spectrum. The color images show the amplitude of the radial component of the Poynting vector in the middle plane normal to the NW axis. Inset: spectral absorption enhancement by a back-reflector for the planar cell and the *R* = 50 nm NW cell.

From the above discussion, a clear understanding of the light-absorption mechanism in a single standing NW is needed to make full use of the built-in concentration effect and achieve the highest *V*_oc_. For this purpose, we define the absorption efficiency *Q*_abs_ as the ratio of the absorption cross-section and the geometrical cross-section of the NW. In Fig. 2(a) and (b), *Q*_abs_ is displayed as a function of the NW radius *R* and the incident wavelength *λ* for a single standing NW on a transparent substrate and on a perfectly reflecting back-reflector, respectively. Several resonance peaks are clearly visible in both cases, dominated by the one with the smallest radius. Compared to the ones analyzed by Cao *et al.* for a lying NW^6^,^7^, the patterns of *Q*_abs_ are similar in shape. However, there are some crucial differences. First, Cao *et al.* employed the Lorentz-Mie scattering formalism, where the NW is treated as an infinitely long cylinder and the absorption efficiency *Q*_abs_ is expressed as the sum of an infinite series. An *n*th-order leaky-mode-resonance (LMR) occurs when the denominator *Δ*_n_ of the *n*th term goes to 0^13^, *i.e.*

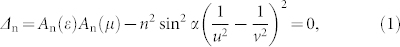
where 
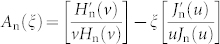
, and *α* denotes the incident angle (see Ref. 18 for the definition of other parameters). The above equation is equivalent to the one given by Cao *et al.*, and determines the LMR peak positions as discrete values of 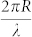 for incident angle 0 < *α* < 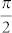. However, for a standing NW where *α* = 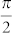, this method predicts the lowest order (*n* = 1) resonance at 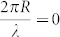, which clearly differs from the simulated results or experimental observations. Since the condition *Δ*_n_ = 0 also represents the cutoff frequency of an infinitely long cylindrical dielectric waveguide (which is 0 for the lowest mode HE_11_)^12^, it is most likely that the discrepancy arises from viewing the standing NW as infinitely long. Second, any resonance peak determined by equation (1) should show up as a straight line through the origin in the *Q*_abs_(*λ*,*R*) plot. While it is roughly true for a lying NW, the peak positions in Fig. 2(a and (b)) deviate more notably from this description. Third, a shift in peak position is observed for a standing NW on a back-reflector compared to that on a transparent substrate (for instance, the maximum of *Q*_abs_ is found at *λ* = 610 nm for a NW with *R* = 50 nm, whereas it shifts to around *λ* = 630 nm for the same NW radius when a back-reflector is applied), which can hardly be explained quantitatively or even qualitatively under the Lorentz-Mie scattering formalism.

Here we present a thorough discussion on the absorption mechanism within a single standing NW. With a finite length, a standing NW is essentially a DRA that supports a series of size-dependent modes by trapping light within its boundaries through internal reflection^8^,^9^. For a cylindrical DRA, these modes can be divided into the transverse-electric modes (TE_0*mp* + *δ*_), the transverse-magnetic modes (TM_0*mp* + *δ*_), and the hybrid modes (HE*_nmp_*_ + *δ*_/EH*_nmp_*_ + *δ*_), where the subscripts *n*, *m*, and *p* denote the azimuthal variation of the fields, the order of variation of the field along the radial direction, and the order of variation of fields along the z-direction (δ denotes an incomplete period), respectively^8^,^9^. When illuminated by a plane wave in the axial direction (which carries only an *n* = 1 component), only the *n* = 1 HE modes are effectively excited in the DRA, with the lowest-order one HE_11δ_ carrying most of the energy^8^,^9^. Systematic studies have been done to determine the resonant frequencies of an arbitrary DRA. For a cylindrical one, although a simple analytical equation is not available (like equation (1)), there are closed-form expressions for the resonant frequencies based on the numerical results of rigorous methods. Mongia *et al.*^9^ have summarized such expressions for the lowest modes that are widely used in the field of DRA, namely (for the HE_11δ_ mode) 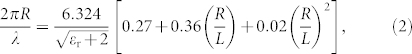
where *R* denotes the DRA radius, *λ* denotes the resonant wavelength, *ε*_r_ denotes the relative permittivity and *L* denotes the DRA length. The resonant condition predicted by equation (2) is shown as the dashed white line in Fig. 2(a), where we have taken *ε*_r_ = 11.9 (a-Si) and *L* = 500 nm. An excellent agreement is found between the simulated peaks of *Q*_abs_ and the predicted HE_11δ_ mode resonance. This is a clear evidence that the absorption of a single standing NW is dominated by the coupling of the incident light into the DRA modes, and the NW functions as a most effective antenna (with a highest *Q*_abs_) when the incident wavelength matches one of its supported modes and causes a leaky-mode-resonance. The literature has not provided an expression for the second lowest mode HE_12δ_ designated in Fig. 2(a), since these higher-order modes are only weakly excited and are less important for antenna applications^8^,^9^ (which is consistent with the simulated results: when the HE_11δ_ mode is excited, the optical cross-section is enlarged to over 17 times its geometrical cross-section, whereas for the HE_12δ_ or higher-order modes, this number is below 5). To further verify the validity of this interpretation, we have compared the simulated results in Ref. 3 for a 2.5 μm long standing GaAs NW with the resonant condition given by equation (2) (where we have taken *ε*_r_ = 13.18 (GaAs), *L* = 2.5 μm). A remarkable match is also found between the HE_11δ_ mode and the lowest-branch (left most) resonance in the absorption cross-section (note that this paper presents the absorption cross-section, for which the second branch HE_12δ_ dominates in magnitude. When normalized to the geometrical cross-section, the lowest branch HE_11δ_ shows the highest *Q*_abs_ and thus the strongest antenna effect, in agreement with our previous discussion). Since their simulated results have been proven by experimental measurements, we believe the DRA model describes the absorption mechanism of a single standing NW quite accurately. It also suggests that the promotion of the open-circuit voltage and the apparent efficiency beyond the Shockley-Queisser limit would be more significant if the NW diameter can be further reduced from ~425 nm to ~250 nm (to match the HE_12δ_ mode within the main absorption spectrum) or eventually to ~100 nm (to match the HE_11δ_ mode within the main absorption spectrum, which would produce the highest built-in concentration).

Compared to the Lorentz-Mie scattering formalism, the DRA model not only gives the right prediction of the dominant resonance peak in a standing NW, it also well explains the influence of a back-reflector. When a metallic plane is introduced below a DRA, this new structure is equivalent to an isolated DRA of double the height under the condition that the symmetric plane is an electric wall (*E*_z_ = 0 on this plane) for the specific mode^9^. This condition is satisfied for all HE modes in a cylindrical DRA, so a standing NW of length *L* on a perfect back-reflector is equivalent to an isolated NW of length 2 *L* under axial illumination (where only the HE modes are excited). In Fig. 2(b), we have plotted the HE_11δ_ mode as a dashed white line by taking *ε*_r_ = 11.9 (a-Si) and *L*' = 2 *L* = 1000 nm. A good agreement is found between the *Q*_abs_ of a 500 nm-long NW on a back-reflector and the HE_11δ_ mode of a 1000 nm-long isolated NW, whereas the HE_11δ_ mode of a 500 nm-long isolated NW shown in Fig. 2(a) distinctly deviate from the *Q*_abs_ plot in Fig. 2(b). This precise prediction of the peak-shifting in *Q*_abs_ strongly suggests that a standing NW is indeed a DRA that absorbs light energy by coupling the incident light into its DRA modes. A similar phenomenon is also observed for the HE_12δ_ mode, although further study is needed to quantitatively prove its origin.

Another important effect of the back-reflector is revealed by comparing the *Q*_abs_ magnitude in Fig. 2(a) and (b). When the HE_11δ_ mode is excited, a NW with a back-reflector shows a *Q*_abs_ of ~35, over twice the value of the same NW on a transparent substrate. More generally, Fig. 2(b) exhibits an approximately 2-fold increment in *Q*_abs_ for all NW radius and incident wavelength compared to Fig. 2(a). This surprisingly high gain in the absorption of a nano-scale device with a back-reflector has been experimentally demonstrated by Kempa *et al.*^2^, where they observed an almost doubled *J*_sc_ of a coaxial NW when a back-reflector is applied. This phenomenon is quite important for our discussion since the additional 2-fold increment in *Q*_abs_ would lead to a more pronounced built-in concentration effect and a higher *V*_oc_ above the planar limit. In Fig. 2(c), we integrate the *Q*_abs_ from Fig. 2(a) and (b) with the AM1.5 solar spectrum and plot the resulting *J*_sc_ for the case without (solid blue line) and with (solid red line) a back-reflector, respectively (assuming an IQE of unit). Apparently, the back-reflector effectively promotes the absorption in a standing NW, leading to the highest built-in concentration *C* = 21 for a NW of radius *R* = 50 nm, whereas the same NW shows only *C* = 10 on a transparent substrate. The more than doubled built-in concentration suggests a fundamental difference between the way a back-reflector promotes the absorption in a planar cell and the way it does in a NW cell. This is revealed in the inset of Fig. 2(c), where the absorption enhancement ratio (defined as the ratio of *Q*_abs_ with and without a back-reflector for a NW cell of *R* = 50 nm, and the ratio of *J*_sc_ with and without a back-reflector for a planar cell) is plotted against the incident wavelength. For a planar cell, the back-reflector promotes the absorption by reflecting the transmitted light back into the cell and prolongs its optical path, thus the promotion can only be seen for longer wavelengths where the material is relatively weak-absorbing, as indicated in the inset of Fig. 2(c). By sharp contrast, the NW cell benefits from the back-reflector for all wavelengths. This originates from the fact that LMR plays a crucial role in the absorption of a nano-scale device^6^, and the effectiveness of the excitation of such leaky modes strongly depends on the overlap between the source field and the mode field. As evidenced in the above discussion, a standing NW on a back-reflector is equivalent to an isolated NW with double the length. Thus, the leaky modes in this new prolonged antenna have a doubled space-overlap with the external driving field and are more effectively excited. We have given an intuitive illustration in the color images where the amplitude of the radial component of the Poynting vector is plotted for a NW of *R* = 50 nm with and without a back-reflector, respectively. This quantity is a straight-forward measurement of the coupling efficiency, and it clearly shows that a NW with a back-reflector is a much more effective antenna.

**Figure 3:**
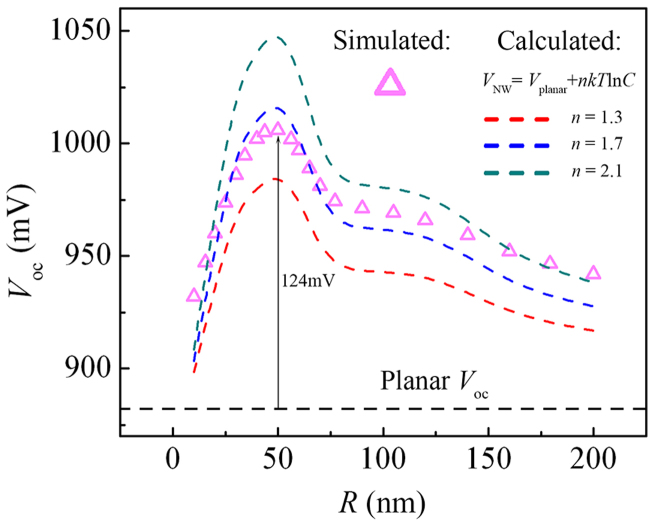
Simulated *V*_oc_ versus *R* for NWs on a back-reflector. The dashed color curves show the calculated results by using *V*_NW_ = *V*_planar_ + *nkT*ln*C*, for *n* = 1.3, 1.7, and 2.1, respectively.

So far, we have thoroughly discussed the optical absorption of a single standing NW. We will now determine its influence on the cell's open-circuit voltage. In the following, we will only consider the cases with a back-reflector (corresponding to Fig. 2(b), or the red solid line in Fig. 2(c)), since they generally have higher built-in concentrations. In Fig. 3, the hollow triangles show the simulated *V*_oc_ of a NW cell with varying radius, while the dashed black line shows the *V*_oc_ of the planar counterpart under the total-absorption condition. The planar cell exhibits a *V*_oc_ of 882 mV, in close proximity to that of the world-record a-Si solar cell (877 mV)^14^, proving the validity of the simulation models. Compared to the planar limit, the NW cells show substantially higher open-circuit voltages, with a maximum of 1.006 V observed for a NW cell with *R* = 50 nm. The 124 mV increment in *V*_oc_ clearly proves that a NW cell is capable of operating beyond the Shockley-Queisser limit. To quantitatively relate this enhancement to the built-in concentration effect, we have taken the general formula for concentrated photovoltaics: *V*_NW_ = *V*_planar_ + *nkT*ln*C*, and plotted three sets of estimated values of *V*_NW_ for different diode quality factors *n* = 1.3/1.7/2.1, respectively (here *V*_NW_ and *V*_planar_ denote the open-circuit voltages of the NW cell and the planar cell, respectively. And *C* is the concentration factor defined as the ratio of *J*_sc_ between the NW cell and the planar cell). Apparently, there is a direct correlation between the extent to which the *V*_oc_ of the NW cell exceeds the planar limit and the logarithm of the built-in concentration *C*, and the cell can be viewed as having a quality factor *n* = 1.5 ~ 2 under illumination, quite consistent with the empirical values for a-Si solar cells^15^. Thus, it is evident that the built-in concentration effect of a standing NW promotes its open-circuit voltage beyond the one-sun planar limit in an almost identical way as an external concentrator applied to a planar cell. Finally, we should point out that a higher open-circuit voltage directly translates to a higher energy-conversion efficiency of the NW cells. This extra efficiency finds its origin in the fundamental process of energy-conversion in a solar cell, where the solar radiation is converted in to an electron-hole gas inside the cell with a chemical potential *μ* given by 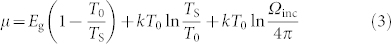
here *E*_g_, *T*_0_, *T*_S_, and *Ω*_inc_ denote the band gap of the cell material, the temperature of the cell, the temperature of the sun, and the receiving solid angle of the solar radiation, respectively^16^. Any macro-scale solar cell without external concentration has an *Ω*_inc_ = *Ω*_s_ = 6.8 × 10^−5^ (the solid angle of the sun), resulting in the Shockley-Queisser limit of conversion efficiency. However, a standing NW cell is able to interact with the solar radiation in a solid angle much larger than *Ω*_s_ owing to its optical antenna effect, thus the entropy loss in the energy transfer is suppressed and the cell shows up as a more efficient thermal electronic engine^17^.

### Carrier-extraction capability beyond the planar limit

In the above section, we have discussed the distinctively different optical-absorption mechanisms in a planar cell and in a standing NW cell, the latter of which is dominated by the coupling of light into the cell's DRA modes. In this section, we will show that such difference not only results in an elevated chemical potential *μ* of the electron-hole gas generated within the cell (which translates to a higher *V*_oc_), but also leads to a more effective extraction of these photocarriers, embodied by a notable rise in the IQE. To begin with, we briefly go through the carrier-extraction of a solar cell. When an electron-hole pair is excited, they must be diverted to their respective contacts before recombination to contribute to the current. The efficiency of this process is measured by IQE, defined as the ratio of collected carriers and generated carriers. In practice, IQE is less than unit to an extent determined by the cell construct, the optical-absorption profile, and the density of states of the material, *etc*. While the last factor can be remedied by improving the quality of the material, there is an inherent difficulty in coordinating the first two factors to achieve an optimal cell performance^4^,^15^,^18^. This is reflected by the fact that the optical-absorption in any macro-scale solar cell submits to an exponential distribution, where most of the photocarriers are generated near the front surface. Nevertheless, the same region often resides the heavily-doped defect-rich emitter, leading to an inevitable recombination loss. Such conflict between the absorption region and the electrical structure accounts for a critical part of current loss in many types of solar cells and puts an extra constraint on cell designs. However, a standing NW cell is not bound by this limit due to its distinct light-absorption mechanism. In this case, the absorption region is primarily defined by the field profile of the excited modes. Thus, it offers an additional degree of freedom in cell designs and can potentially improve the cell efficiency.

**Figure 4:**
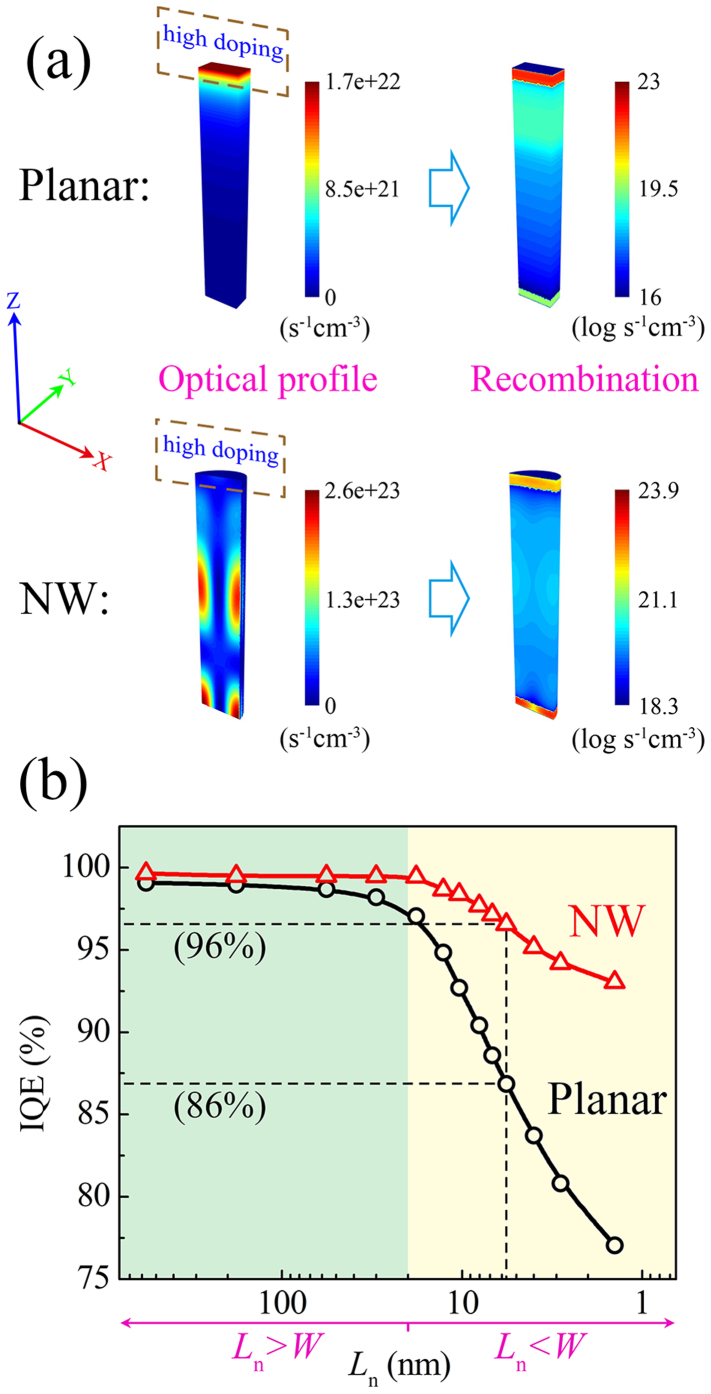
(a) Carrier generation profile and logarithmic recombination rate within the planar cell and the *R* = 50 nm NW cell on a back-reflector. The dashed frames show the region of the heavily-doped emitters. (b) IQE of the planar cell and the *R* = 50 nm NW cell on a back-reflector for different diffusion lengths *L*_n_ in the doped layers. *W* is the thickness of the doped layers and has a constant value of 20 nm.

For consistency, we will illustrate this effect based on the cell structure with the highest open-circuit voltage determined in the previous section, namely the *R* = 50 nm NW cell on a back-reflector. We will see later that this choice is well-founded by the simultaneous dependence of both the promotion in *V*_oc_ and IQE on the excitation of the HE_11δ_ mode. In the left part of Fig. 4(a), the optical-absorption profile under AM1.5 illumination is plotted as color maps for the planar cell and the *R* = 50 nm NW cell on a back-reflector, respectively (the numbers correspond to the carrier-generation rate). The region of the heavily-doped p-type emitter is also labeled by dashed frames in both cases for comparison with the optical profile. Apparently, the absorption in the planar cell exhibits a rapid exponential decay along the Z-minus axis due to the dominant role of the middle-wavelength light in the AM1.5 solar spectrum, for which the a-Si material is quite absorptive. As a result, a large overlap is found between the place of generation of the photocarriers and the region of the heavily-doped emitter, implying a considerable current loss (as will be shown in the following). By contrast, the absorption pattern in the NW cell is distinctively different. Its photocarriers are concentrated around specific locations along the axial direction, with a clear azimuthal symmetry. According to the previous discussion, this is a typical mode profile of the HE_111 + δ_ mode excited by *λ* = 630 nm, where the azimuthal variation of the fields is of the form sinϕ (thus *n* = 1), the radial variation of the fields has one maximum (thus *m* = 1), and the axial variation of the fields has one complete period (thus *p* = 1). Unlike the planar case, the optical profile in the NW cell cannot simply be viewed as a superposition of the respective profiles of different wavelengths weighted to their intensity in the AM1.5 solar spectrum (otherwise we should anticipate a much less characterized optical pattern due to the contribution from the off-resonance wavelengths that dominate in intensity). Instead, the highly characterized HE_11δ_ mode profile results predominantly from the optical antenna effect, where the NW responds to the resonant wavelength (*λ* = 630 nm) with a much larger cross-section than the off-resonance ones and thus exhibits field patterns close to that of a single mode. This is particularly beneficial to the cell designs since it provides us with a simple and effective control over the absorption profile within the cell. Finally, the shifting of the absorption region from the heavily-doped surface layer to the intrinsic middle layer clearly solves the dilemma faced by the planar cell, as is shown in the following.

In the right part of Fig. 4(a), the color maps show the logarithmic recombination rate within the planar cell and the *R* = 50 nm NW cell on a back-reflector, respectively, under AM1.5 solar spectrum. We have used the exact same set of electrical parameters in these simulations to reveal the exclusive influence of an altered absorption profile. The resultant IQE (integrated value for the whole AM1.5 spectrum, the same below) rise is remarkable: from 86% for the planar cell to an extremely high value of 96% for the NW cell, which corresponds to a 3.5-fold reduction in recombination-induced current loss. The origin of such enhancement is found by comparing the magnitude of the recombination rate in these two cells. The recombination rate in the heavily-doped emitter is one order of magnitude higher in the planar cell than in the NW cell, despite the fact that the latter carries a current density 20 times higher than the former (due to the built-in concentration). This is a direct embodiment of the ineffectiveness of the planar cell design regarding carrier-extraction. On the other hand, the suppressed recombination in the emitter of the NW cell comes at the expense of a 3 ~ 4 order-of-magnitude increase in the recombination rate in the i-layer, due to both the localized maximums of the electrical field and the optical antenna effect. This, however, proves to have little influence on the overall carrier-extraction of the NW cell since the intrinsic layer is in nature an ideal absorber of light with a negligible base recombination rate. The dominant recombination loss in the NW cell is still attributed to the surface doped layers, as is evident in the figure. It should be noted that there are several regions in the n-doped bottom layer of the NW cell where the optical maximums partly overlap with the doped region so the local recombination rate is relatively high. Nevertheless, the impact of such overlap is limited by the sinusoidal variation of the optical fields along the Z-minus axis in the HE_11δ_ mode, where only a small proportion of light energy resides in the surface doped layer, in contrast to the exponential case exhibited by the planar cell^9^.

At last, we will give a more general demonstration of the advantage of the standing NW structure in cell designs by comparing the carrier-extraction capability of the planar cell and the NW cell in different parameter ranges. The minority carrier diffusion lengths in the doped layers, *L*_n_(electrons) and *L*_p_(holes), are critical indicators of the cell performance and are given prior consideration during the fabrication of the a-Si solar cells. We have varied their values from a default *L*_n_/*L*_p_ = 6.3 nm/5.8 nm to a range of 1 nm ~ 600 nm by changing the dopant concentration in the p-layer/n-layer simultaneously (*L*_n_ and *L*_p_ are kept proportional and in close values in all cases and only *L*_n_ is later shown as the variable). In Fig. 4(b), the IQEs of the planar cell and the NW cell are shown for two distinct regions: (1) *L*_n_ > *W* (light green); and (2) *L*_n_ < *W* (light yellow), where *W* is the thickness of the doped layers and has a constant value of 20 nm. This division follows the approximate rule of solar cells that the photocarriers can be effectively collected only when their diffusion lengths are equal to or larger than the respective layer thicknesses. It is apparent from the figure that both the planar structure and the standing NW structure can efficiently extract the photocarriers when the diffusion lengths in the doped layers are sufficiently long (*L*_n_ > *W*), embodied by IQE values close to unity in the left region. However, such a condition requires an excellent quality of the material and puts a strict constraint on the doping concentrations, which is of limited interest to practice use. The superiority of the standing NW structure lies in the right region where *L*_n_ < *W*, corresponding to the actual cases where the carrier diffusion lengths within the doped layers are often limited to a few nanometers^18^,^19^. In this region, the NW structure is able to maintain a relatively high carrier-collecting efficiency (IQE > 90%) even for diffusion lengths small than 1 nm, while the planar structure suffers dramatically with decreasing *L*_n_, showing an IQE of ~75% at *L*_n_ = 1 nm. Such difference, as is discussed above, has arisen from the fact that the absorption front in the NW cell is shifted from the doped emitter to the intrinsic middle layer when its HE_11δ_ DRA mode is excited, which provides the NW cell with inherent tolerance to doping-induced recombination. The congruity between the electrical structure and the absorption profile of the NW cell opens up many new potentials in cell designs: ultra-high doping and much thicker emitters can be used to meet the requirements for a strong built-in electric field or a high-quality contact, the restriction on the deposition technique is loosen, and the current loss in the intrinsically defect-rich materials can be suppressed when applied to certain types of heterojunction solar cells, *etc*. Finally, it is intriguing to compare these theoretical results to those of the latest experimental investigations. In the work by Wallentin *et al.*^4^, the EQE of the standing-NW-array InP solar cell is found to sensitively depend on the thickness of the top n-segment, in contrast to our previous findings. As an explanation, they showed by optical simulation that most of the photocarriers are generated near the top of the NWs under the chosen NW dimensions. This discrepancy has most likely arisen from the fact that the NW diameter in their work (180 nm) is too large to support the HE_11δ_ mode resonance within the main absorption spectrum. In such a case, the superposition of the optical profiles of the off-resonance wavelengths would lead to a non-characteristic planar-like absorption pattern (as is evidenced by their simulation) and diminishes its advantage in quantum efficiency. It would be helpful to examine the cell performance based on standing NWs with smaller diameters (~100 nm), for which the superior carrier-extraction capability should be clearly demonstrable.

## Discussion

**Figure 5:**
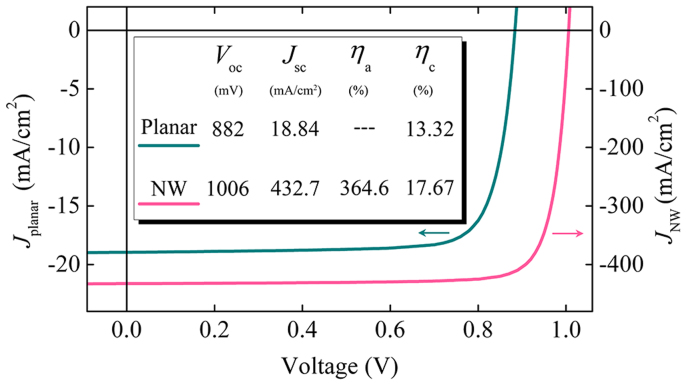
*I*–*V* characteristics and cell parameters of the planar cell and the *R* = 50 nm NW cell on a back-reflector. The apparent efficiency *η*_a_ is calculated with the projected area of the NW cell.

The previous sections have assessed two different approaches in efficiency improvement regarding open-circuit voltage and internal quantum efficiency, respectively. The efficiency gain in both cases relies directly on the excitation of the HE_11δ_ resonant mode, which indicates that the NW diameter should be chosen according to the resonant condition of the main absorption wavelength in order to achieve maximal conversion efficiency. Experimentally, this choice can be enabled by many techniques such as vapor–liquid–solid (VLS) growth and metal-assisted chemical etching (MACE), which are capable of fabricating NWs with diameters as small as 10 nm^20^. Also, the use of a back-reflector can further promote the effects. Here in the following, we give an overall comparison between the performance of the planar cell and that of the optimal standing NW cell. The simulated *I*–*V* curves of the planar cell (dark green, under total-absorption condition) and the NW cell (light purple, *R* = 50 nm with a back-reflector) are shown in Fig. 5 and their cell parameters are listed in the middle chart. The open-circuit voltage and the short-circuit current density of the planar cell shows upper limits of 882 mV and 18.84 mA/cm^2^, respectively, in good accordance with the experimental records for a-Si solar cells^14^. By comparison, the NW cell shows a *V*_oc_ 124 mV beyond the planar limit due to the built-in concentration, and exhibits an extremely high *J*_sc_ of 432.7 mA/cm^2^ under the combined influence of the built-in concentration and the promoted carrier-extraction efficiency. When calculated with the projected area of the cell (which is the horizontal cross-section of the NW), the apparent efficiency (*η*_a_) of the NW cell is 364.6%, several times higher than the ray-optics limit. Such a high *η*_a_ shows that the standing NW cell can generate a considerable amount of electric work while occupying a very small footprint, which makes it a perfect choice for nano-scale electric power sources on a chip or for sensor applications^3^. Finally, and most importantly, the energy conversion efficiency (*η*_c_, defined as the ratio of the output electric energy and the absorbed photon energy) of the NW cell reaches a high value of 17.67%, showing a 33% relative increase compared to the planar cell limit (13.32%, which is close to the initial efficiency of the record a-Si cell in experiments). This improvement in energy-conversion efficiency is attributed to the fact that the NW cell is able to convert the solar radiation into an electron-hole gas with a higher chemical potential, and that it collects these photocarriers more effectively due to the congruity between its electrical structure and optical profile. Therefore, the standing NW structure could be a promising building block for ultrahigh-efficiency solar cells or a platform for high efficiency nano-scale energy sources.

## Methods

The left part of Fig. 1(a) shows the cell structure of the simulated single a-Si standing NW solar cells. A typical p-i-n structure of the a-Si thin film solar cells is adopted, namely (from top down): the front TCO contact (omitted in the figure), the p-doped a-Si layer, the intrinsic a-Si layer, the n-doped a-Si layer, the rear TCO contact (omitted in the figure), and the substrate (a transparent dielectric with a refractive index *n* = 1.5 for the cases without a back-reflector, or a perfectly reflecting metal for the cases with a back-reflector). To highlight the potential of the single standing NW cell to perform beyond the planar limits, both the planar cell thickness and the NW length are kept constant at 500 nm, with a fixed configuration p = 20 nm/i = 460 nm/n = 20 nm^14^,^21^ (the intrinsic layer thickness is relatively enlarged to ensure comparable absorption without light-trapping structure). The NW radius *R* varies from 10 nm to 200 nm.

We employ the finite-difference time-domain (FDTD) method to obtain the light absorption of the cells. The incident light is an infinite plane wave directed to the minus Z-axis (which is normal incidence for the planar cell and axial incidence for the NW cell), with a wavelength *λ* ranging from 300 nm–720 nm, covering the major absorption spectrum of a-Si (*E*_g_ = 1.72 eV). The simulations are performed in a 200 nm × 200 nm × 1 μm FDTD region with periodic boundary conditions in the in-plane directions for the planar cell, and in a 4 μm × 4 μm × 1 μm region with perfectly matched layer (PML) boundaries in the in-plane directions for the single standing NW cells (further increasing the latter region size in the x-y plane results in little change of the absorption within the NW, thus this setup offers a good approximation of an isolated NW under infinite axial illumination). A mesh-refinement of down to 1 nm is applied and the complex refractive indices *n*, *k* of the material are taken from a widely used reference^22^ to ensure the credibility of the simulated optical fields. To assess the upper limit of the planar cell performance, the simulated planar absorption rate *Abs*(*λ*) is artificially divided by a factor of [1-*R*(λ)-*T*(λ)] prior to the subsequent electrical simulations, where *R*(*λ*) and *T*(*λ*) are the reflectance from and the transmittance through the cell, respectively. This corresponds to the total-absorption condition of the planar cell and gives an upper limit of photocurrent.

For electrical simulations, basic semiconductor equations are solved on a tetragonal mesh with a fine mesh-size of 1 nm. The photocarrier generation rate at each mesh point is given by integrating *Abs*(λ) with the AM1.5 solar spectrum in the interval of 300 nm–720 nm. The top and rear contacts are assumed to be ohmic while the a-Si is characterized as a semiconductor material with a band gap of 1.72 eV^19^. Both the Shockley-Read-Hall (SRH) recombination and Auger recombination are considered in each layer, giving an electron diffusion length of ~500 nm in the i-layer and ~6 nm in the doped layers^14^,^19^,^21^. For simplicity, the influence of the interface/surface recombination and the doping concentration on the NW cell^23^,^24^ is not discussed, which does not affect the main conclusions of this work. All the above simulations are performed using a commercial software package [FDTD Solutions v8 & DEVICE v3, Lumerical 2013], the validity of which has been proven by numerous works regarding nano-scale optoelectronic devices.

## Author Contributions

Y.Z. carried out the simulations. Y.Z., Q.Y. and W.S. contributed to the data analysis and figures. Y.Z. wrote the main manuscript text and W.S. reviewed the manuscript.
